# Placenta histopathology in SARS-CoV-2 infection: analysis of a consecutive series and comparison with control cohorts

**DOI:** 10.1007/s00428-021-03097-3

**Published:** 2021-05-01

**Authors:** Luca Bertero, Fulvio Borella, Giovanni Botta, Andrea Carosso, Stefano Cosma, Marialuisa Bovetti, Marco Carosso, Giancarlo Abbona, Giammarco Collemi, Mauro Papotti, Paola Cassoni, Chiara Benedetto

**Affiliations:** 1 Pathology Unit, Department of Medical Sciences, University of Turin and “Città della Salute e della Scienza di Torino” University Hospital, Turin, Italy; 2 Obstetrics and Gynecology 1U, Department of Surgical Sciences, Sant’Anna Hospital, University of Turin and “Città della Salute e della Scienza di Torino” University Hospital, Turin, Italy; 3 Pathology Unit, “Città della Salute e della Scienza di Torino” University Hospital, Turin, Italy; 4 Pathology Unit, Department of Oncology, University of Turin and “Città della Salute e della Scienza di Torino” University Hospital, Turin, Italy

**Keywords:** COVID-19, SARS-CoV-2, Histopathology, Thrombosis, Inflammation, Obstetrics

## Abstract

Infection by SARS-CoV-2 has been shown to involve a wide range of organs and tissues, leading to a kaleidoscope of clinical conditions. Within this spectrum, an involvement of the fetal-maternal unit could be expected, but, so far, the histopathological evaluation of placentas delivered by women with SARS-CoV-2 infection did not show distinct hallmarks. A consecutive series of 11 placentas, delivered by 10 women with COVID-19 admitted to our Obstetrics and Gynecology clinic have been investigated and compared to a control cohort of 58 pre-COVID-19 placentas and 28 placentas delivered by women who had a previous cesarean section. Four out of eleven placentas showed changes consistent with chronic villitis/villitis of unknown etiology (VUE), while in one case, chronic histiocytic intervillositis was diagnosed. Thrombo-hemorrhagic alterations were observed in a subset of cases. Compared to the control cohort, chronic villitis/VUE (*p* < 0.001), chronic deciduitis (*p* = 0.023), microvascular thrombosis (*p* = 0.003), presence of infarction areas (*p* = 0.047) and of accelerated villous maturation (*p* = 0.005) showed higher frequencies in placentas delivered by women with COVID-19. Chronic villitis/VUE (*p* = 0.003) and accelerated villous maturation (*p* = 0.019) remained statistically significant by restricting the analysis to placentas delivered after a previous cesarean section. The observed differences in terms of pathological findings could be consistent with SARS-CoV-2 pathogenesis, but just a subset of alterations remained statistically significant after adjusting for a previous cesarean section. A careful consideration of potential confounders is warranted in future studies exploring the relationship between COVID-19 and pregnancy.

## Introduction

On March 11, 2020, due do the rapid escalation of the coronavirus disease 2019 (COVID-19) outbreak, the World Health Organization (WHO) declared a pandemic [61]. COVID-19 is caused by the severe acute respiratory syndrome coronavirus 2 (SARS-CoV-2), a betacoronavirus similar to SARS-CoV and MERS-CoV, with multiple possible transmission routes and characterized by a high infectivity [59, 62].

In terms of maternal outcomes, data derived from initial cohorts suggested that COVID-19 clinical course in this group of patients is similar to non-pregnant women [11, 16, 17, 23, 28, 40], but not all data are reassuring. Seven maternal deaths were reported among nine pregnant women with severe COVID-19 [32], and an analysis of a Swedish cohort suggested an increased risk of being admitted to an intensive care unit [relative risk (RR): 5.4; 95% Confidence Interval (CI) 2.89–10.08] and of receiving invasive mechanical ventilation (RR: 4.0; 95%; CI 1.75–9.14) for pregnant women with COVID-19 compared to non-pregnant women of similar age [19, 60].

The role of SARS-CoV-2 in the first trimester of pregnancy has also been investigated and present data do not suggest an increased spontaneous abortion risk [21], but the long-term consequences are currently unknown and, in the endemic areas, tests for SARS-CoV-2 should be offered to all pregnant women to investigate this open question [20].

Concerning neonatal outcomes, fetal and respiratory distress as well as thrombocytopenia accompanied by abnormal liver function and even death have been described [64]. In addition, evidence of a higher incidence of preterm birth has been reported [25, 29, 63] as well as cases of SARS-CoV-2 vertical transmission [5, 12–14, 26, 58], but this seems to be an uncommon occurrence [24, 25, 63].

High expression of proteins (ACE2/TMPRSS2) required for SARS-CoV-2 cell entry has been observed in maternal-fetal interface tissues [42], and placental infection by SARS-CoV-2 has been demonstrated in a minority of cases [1, 2, 27, 33, 55, 58].

To date, placental histopathology in COVID-19 has been investigated [6, 7, 18, 22, 31, 33, 47, 48, 53, 54], but specific features or hallmarks have not been identified. Nevertheless, the inflammatory activation and the increased thrombotic risk described in patients with COVID-19 [35, 52, 57] make the placenta a potential target of pathophysiological phenomena which could affect pregnancy outcomes.

The aim of this work was to evaluate a consecutive series of placentas delivered at our institution from women with COVID-19 infection and compare these data with a pre-COVID-19 control series.

## Methods

Our study is based on the pathological analysis of 11 consecutive placentas delivered from 10 women affected by COVID-19 and admitted to the Obstetrics and Gynecology clinic of S. Anna Hospital – “Città della Salute e delle Scienza di Torino”, University of Turin from March 22, 2020 to July 17, 2020. Sample collection was performed within the framework of the “SARS-CoV-2 infection during pregnancy and puerperium: an Italian Obstetric Surveillance System (ItOSS)” study by the Italian National Institute of Health. All patients showed symptomatic COVID-19 disease confirmed by a nasopharyngeal swab according to World Health Organization (WHO) guidelines. SARS-CoV-2 RNA was detected using an automated real-time RT-PCR assay [DiaSorin Molecular Simplexa™ COVID-19 Direct, target genes S and ORF1ab]. All placentas and newborns were tested for SARS-CoV-2 with the same assay while a rectal swab was only performed on the patient whose infant resulted positive to SARS-CoV-2 to investigate the potential route of infection. Pathological examination was performed according to routine procedures and immunohistochemistry was set up on a BenchMark ULTRA platform (Ventana Medical Systems Inc., Tucson, AZ, USA).

Clinical records of our Pathology unit (“Città della Salute e della Scienza” University Hospital, Turin, Italy) were searched to select a control cohort. Placentas submitted to histopathological examination between March 2019 and July 2019 were screened and cases without significant maternal comorbidities (e.g., hypertension, diabetes mellitus, infections during pregnancy) were collected (*n* = 60). Two cases with a gestational age <30 weeks were excluded to match the groups for this parameter. Since the rate of previous cesarean sections was significantly higher in the COVID-19 group, control cases with this characteristic and without significant maternal comorbidities or abnormal placental implantation were selected to define a further control group. To reach a 4:1 control to cases ratio for this group, the search was expanded to the same months of 2018.

The study was conducted in accordance with The Code of Ethics of the World Medical Association (Declaration of Helsinki and following amendments) for studies involving humans and within the guidelines and regulations defined by the Research Ethics Committee of the University of Turin. Patients signed a written informed consent. Data supporting study results are included within the manuscript.

Statistical analyses included the use of the *t*-test for unpaired variables and Fisher’s exact test for comparison of continuous and categorical variables [Stata/MP 15.0 Statistical Software (STATA, College Station, TX, USA)]. Statistical significance was defined as a two-tailed *p* value of <0.05.

## Results

### Case series — general features

The clinical features of the 10 pregnant women affected by COVID-19 and who delivered the 11 analyzed placentas are reported in Table 1. Placental weight percentiles were reported using the nomogram proposed by Almog et al. [3]. No patient had a history of previous thrombosis, systemic autoimmune diseases, recurrent early miscarriages, fetal loss >10 week of gestation, or other conditions suggestive of anti-phospholipid antibody syndrome [30]. All patients resulted negative for the following pathogens: HIV, HBV, HCV, cytomegalovirus, *Treponema pallidum* and *Toxoplasma gondii*. Among the six full-term and the four preterm patients (<37 weeks as defined by the WHO) [8] admitted to the hospital for delivery, one had preterm premature rupture of membranes (pPROM) and one a twin pregnancy with oligohydramnios. Four patients complained of COVID-19 symptoms at the time of delivery, one patient (Patient 1) became symptomatic one day after delivery, one patient (Patient 8) was no longer symptomatic at the time of delivery (COVID-19 symptoms onset at day -95) and the four remaining patients were asymptomatic. All placental swabs were negative for SARS-Cov-2 and only one neonatal nasopharyngeal swab was positive for the virus (as already reported by our group) [12]. In this case, a maternal rectal swab was positive for SARS-Cov-2 suggesting the possibility of a contamination of the newborn during the passage through the birth canal.

**Table 1:** Clinical characteristics of the case series

Case	Age (years)	Previous pregnancies	Gestational Age (weeks)	Comorbidities	COVID-19 symptoms/signs	Symptoms/signs onset (days)*	NP swab date (days)*	PROM	Delivery mode	Indication for C-section	Weight (grams)	Neonatal sex	APGAR scores at 1’ ad 5’	Placental swab	Neonatal nasopharyngeal swab
1	28	1	37	None	Cough, fever	+1	+1	No	Vaginal	/	3120	F	9/9	Negative	Positive**
2	25	2	38	β-thalassemia trait	Fever	−3	0	No	C-section	Two previous C-sections	3010	F	9/9	Negative	Negative
3	30	1	36	None	Cough	−8	−8	No	Vaginal	/	2390	M	9/9	Negative	Negative
4***	35	1	30	None	Fever, rhinorrhea	−11	0	Yes (27 weeks)	C-section	Twin pregnancy (dichorionic/diamniotic), one previous C-section	1090 and 950	F and M	First twin: 5/9 Second twin: 5/9	Negative /Negative	Negative /Negative
5	39	4****	32	Chronic hypertension, severe obesity (BMI: 50), gestational diabetes, polyhydramnios	Rhinorrhea, cough and fever since about one month. At admittance: initial respiratory fatigue with interstitial pneumonia	−39	−9	No	C-section	Respiratory distress, two previous C-sections	2100	F	8/9	Negative	Negative
6	32	1	40	β-thalassemia trait	Asymptomatic	Not applicable	−6	No	Vaginal	/	3530	M	9/9	Negative	Negative
7	37	2	37	None	Asymptomatic	Not applicable	−18	No	C-section	Previous C-section and orthopedic contraindication (spondylolisthesis)	2469	F	9/9	Negative	Negative
8	43	3	37	Intrahepatic cholestasis of pregnancy	Fever, cough	−85	−66	No	C-section	Three previous C-sections and intrahepatic cholestasis of pregnancy	3130	M	9/9	Negative	Negative
9	36	2	35	None	Asymptomatic	Not applicable	−95	No	C-section	Twin pregnancy (monochorionic/diaamniotic), oligohydramnios and previous C-section	2160 and 2060	F and F	First twin: 6/6 Second twin: 8/9	Negative/Negative	Negative
10	32	2	37	None	Asymptomatic	Not applicable	−1	No	C-section	Marginal placenta previa	2700	M	9/9	Negative	Negative

*Day 0 is considered the day of the delivery

**In this case a maternal rectal swab was positive for SARS-Cov-2 suggesting a fecal contamination of the newborn during the vaginal labor

***SARS-CoV-2 infection was found at 27 weeks of gestational age

****A newborn died one month after birth because of causes unrelated to pregnancy (infectious disease)

*NP*, nasopharyngeal; *BMI*, Body Mass Index; *C-section*, cesarean section; *IUGR*, intrauterine growth restriction; *PROM*, prelabor rupture of membranes; *F*, female; *M*, male

**Fig. 1:**
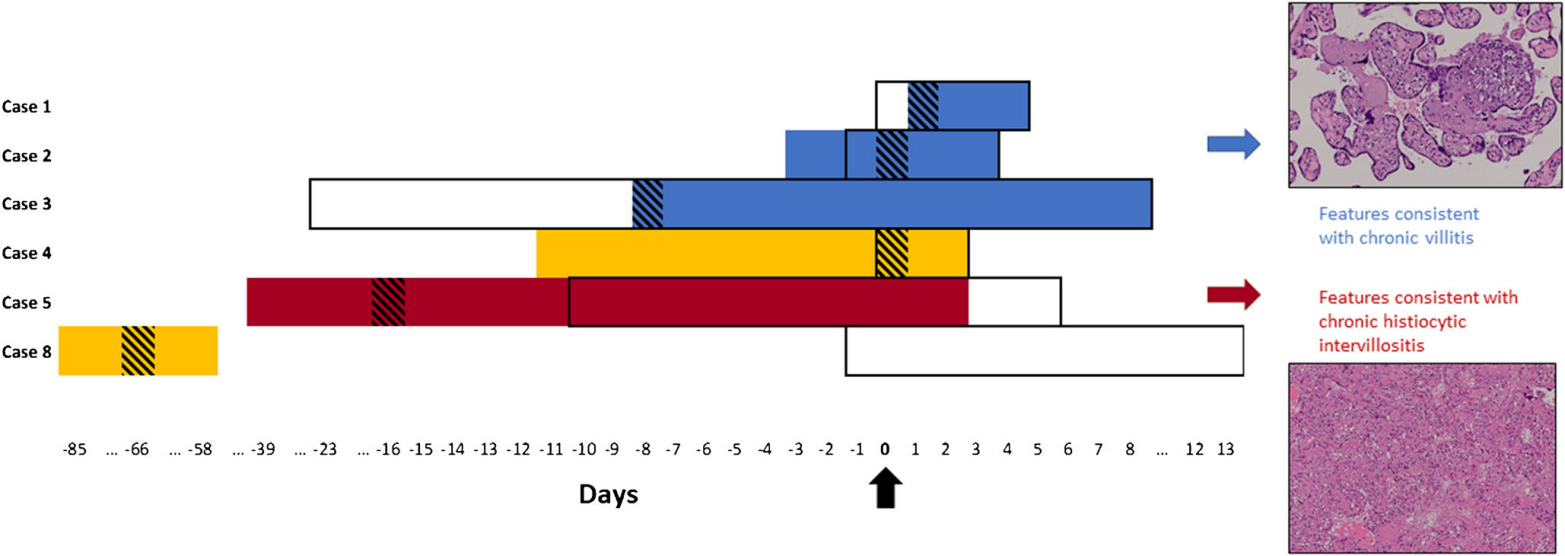
Outline of COVID-19 clinical course in symptomatic patients and placental pathological findings. Findings consistent with chronic villitis were found in patients with shorter COVID-19 duration and milder symptoms (blue), while chronic histiocytic intervillositis was diagnosed in Case 5 after long-standing and more severe symptoms (red). Patient 4 and patient 8 showed unspecific placental findings (yellow). Black arrow (Day 0): delivery time. Black rectangles: hospitalization. Colored boxes: days with COVID-19 symptoms. Striped boxes: time of nasopharyngeal swab

The overall relationship between COVID-19 onset and diagnosis, hospital stay and time of delivery is reported on Fig. 1, while a reference summary of pathological alterations observed in the COVID-19 series is presented in Table 2.

**Table 2:** Pathological findings observed in the COVID-19 case series

Case	Age	Pathological findings
1	28 years	• Weight: 590 g (before fixation), 25^th^–50^th^ percentiles. • Dimensions: 16 cm × 15 cm × 2 cm to 3 cm thickness. • Translucent membranes. • Umbilical cord: 45 cm. Normal morphology and insertion. • Sectioning: no abnormalities. • Scant lymphocytic inflammation (CD8-positive), mainly located within the decidua and the basal half, consistent with chronic villitis (low grade, multifocal). • Microvascular thrombosis in decidual vessels and villi. • Normal villous maturation with just focal signs of hypermaturation.
2	25 years	• Weight: 429 g (before fixation), <3^rd^ percentile. • Dimensions: 15.5 cm × 15 cm × 2 cm to 3.1 cm thickness. • Translucent membranes. • Umbilical cord: 16 cm. Normal morphology. • Sectioning: focal whitish areas close to the chorial surface and small (<1 cm) hemorrhagic foci. • Significant foci of lymphocytic inflammation (CD8-positive) involving the decidua and multiple villi, consistent with chronic villitis (low grade, multifocal). • Microvascular thrombosis in decidual vessels and villi. • Intervillous hematomas. • Normal villous maturation.
3	30 years	• Weight: 370 g (before fixation), <3^rd^ percentile. • Dimensions: 15 cm × 112 cm × 1 cm to 3 cm thickness. • Translucent membranes. • Umbilical cord: 28 cm. Normal morphology and insertion. • Sectioning: focal whitish areas (up to 8 mm). • Villous/intervillous lymphocytic (CD8-positive) inflammation involving the decidua and both the placental maternal and fetal halves, consistent with chronic villitis (low grade, multifocal). • Thrombo-hemorrhagic alterations in decidual vessels, villous infarction, trophoblastic loss and focal thrombi, consistent with decidual vasculopathy and chronic malperfusion. • Normal villous maturation with just focal chorangiosis.
4 First placenta	35 years	• Weight: 410 g (after fixation). • Dimensions: 16 cm × 14 cm × 1 cm to 3 cm thickness. • Greenish discoloration of membranes. • Umbilical cord: 16 cm. Normal morphology and insertion. • Sectioning: extensive subchorial hemorrhagic areas consistent with partial placental abruption. • Histological features consistent with partial placental abruption. • Hemorrhagic areas below the fetal surface, associated with chronic hypoxia signs (intervillous necrotic areas with focal calcifications). • Focal features consistent with accelerated maturation.
4 Second placenta		• Weight: 235 g (after fixation). • Dimensions: 14 cm × 15 cm × 0.5 cm to 2.5 cm thickness. • Greenish discoloration of membranes. • Umbilical cord: 20 cm. Normal morphology and paracentral insertion. • Sectioning: no abnormalities. • Features consistent with accelerated maturation.
		Placental weights were among the 10th–25th percentiles (considering the sum of both placentas for twin pregnancies).
5	39 years	• Weight: 370 g (after fixation), 10^th^–25^th^ percentiles. • Dimensions: 15 cm × 12 cm × 1 cm to 3.2 cm thickness. • Translucent membranes. • Umbilical cord: 18 cm. Normal morphology and paracentral insertion. • Sectioning: focal placental abruption. • Intervillous histiocytic infiltration (CD68-positive) with perivillous fibrin deposition. • Areas with villous conglutination, loss of trophoblasts and focal microcalcifications. • Focal accelerated villous maturation.
6	32 years	• Weight: 490 grams (before fixation), 3^rd^–10^th^ percentiles. • Dimensions: 17 cm × 17 cm × 1 cm to 3 cm thickness. • Translucent membranes. • Umbilical cord: 50 cm. Normal morphology and central insertion. • Sectioning: whitish areas (15 mm). • Irregular villous maturation with focal chorangiosis. • Focal intervillous infarction. • Focal villous calcifications.
7	37 years	• Weight: 400 g (before fixation), <3^rd^ percentile. • Dimensions: 16 cm × 13 cm × 1 cm to 4 cm thickness. • Translucent membranes. • Umbilical cord: 18 cm. Normal morphology and marginal insertion. • Sectioning: focal congestion area. • Widespread chorangiosis. • Avascular villi with sclerosis. • Vascular stenosis and hypertrophy of stem villi. • Findings consistent with umbilical blood flow restriction.
8	43 years	• Weight: 520 g (before fixation), 10^th^–25^th^ percentiles. • Dimensions: 16 cm × 14 cm × 2 cm to 3.5 cm thickness. • Yellowish membranes. • Umbilical cord: 37 cm. Normal morphology and marginal insertion. • Sectioning: no specific alterations. • Focal villous hypermaturation. • Focal villous agglutination.
9	36 years	• Monochorionic diamniotic. • Weight: 665 g (before fixation), 3^rd^–10^th^ percentiles for twin deliveries. • Dimensions: 19 cm × 16 cm × 3 cm to 4.5 cm thickness. • Translucent membranes. • First umbilical cord: 23 cm. Hypocoiled with focal edema and marginal insertion. • Second umbilical cord: 20 cm. Hypocoiled with marginal insertion. • Sectioning: no specific alterations. • Lymphocytic infiltration consistent with chronic villitis (low grade, multifocal). • Accelerated villous maturation associated with focal hypoplasia and increase of syncytial nodes. • Focal villous sclerosis consistent with fetal thrombotic vasculopathy. • Focal hemorrhagic area within basal decidua consistent with recent abruption.
10	32 years	• Weight: 440 g (before fixation), 3^rd^–10^th^ percentiles. • Dimensions: 16 cm × 15 cm × 1 cm to 3 cm thickness. • Translucent membranes. • Umbilical cord: 40 cm. Normal morphology and marginal insertion. • Sectioning: no specific alterations. • Villous hypoplasia. • Focal sclerosis consistent with previous placental infarction.

#### Case 1

Placenta weight was 590 g (25^th^–50^th^ percentiles) before formalin fixation, lateral dimensions were 16 × 15 cm with thickness ranging from 2 cm to 3 cm. Umbilical cord length was 45 cm, its structure normal and a peripheral insertion was observed. Placental macroscopic features were normal with translucent membranes. Histological examination showed only mild abnormalities including a scant lymphocytic inflammation which involved both the decidua and basal placental villi with initial villous agglutination. Lymphocytes showed a prevalent T cell cytotoxic (CD8-positive) phenotype. These findings were consistent with chronic villitis (low grade, multifocal since inflammatory foci were present on more than one slide) [37]. Microvascular thrombosis was observed in decidual and villous vessels. Maturation was normal with features suggesting initial hypermaturation.

#### Case 2

**Fig. 2:**
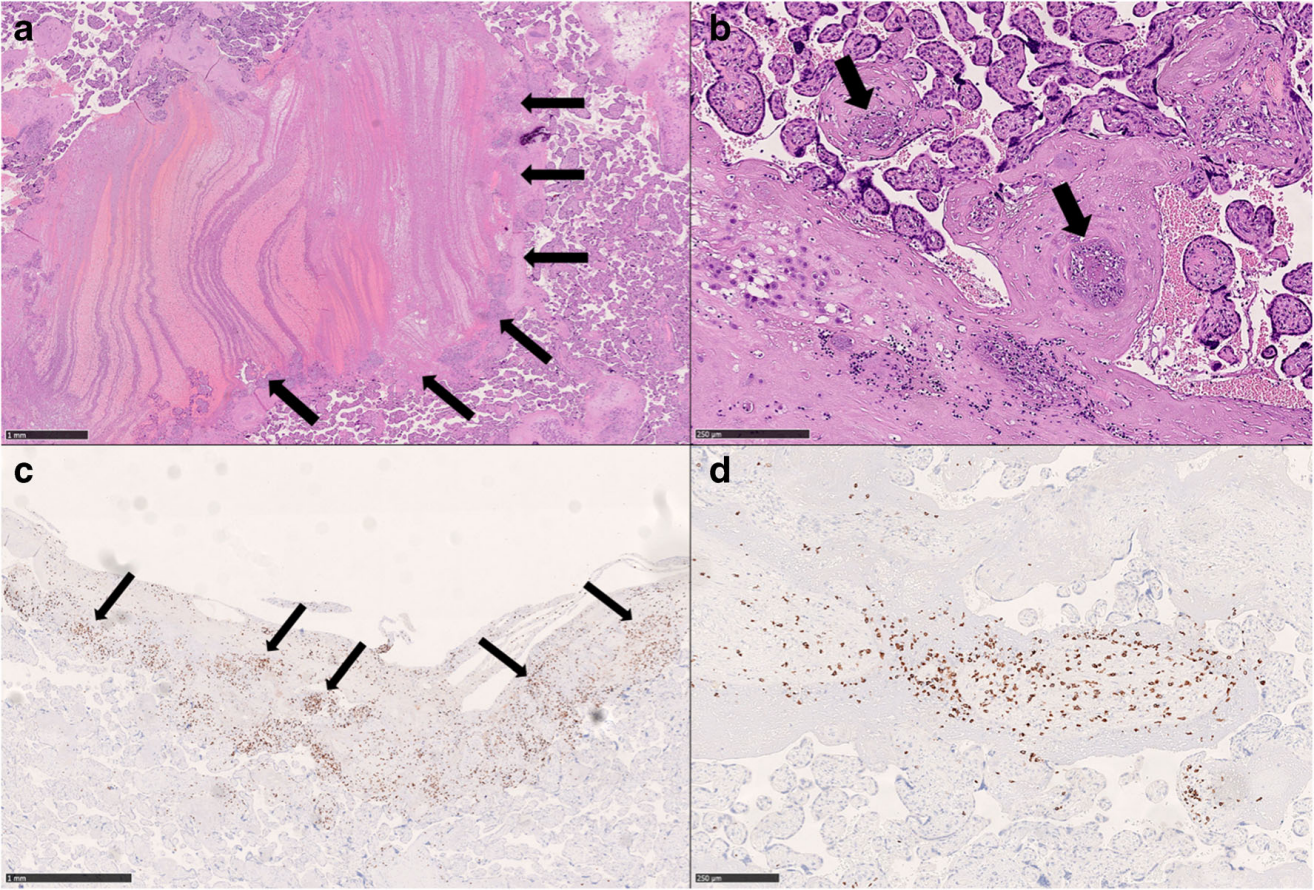
Pathological findings of Case 2. Placental intervillous hematomas (arrows) (**A**: HE, 20X) and microvascular thrombosis (arrows) (**B**: HE, 100X) were observed. Significant foci of decidual and villous inflammation were also present (arrows) (**C**: anti-CD8 IHC, 30X; **D**: anti-CD8 IHC, 80X), consistent with multifocal, low grade chronic villitis

Placenta weighted 429 g (<3^rd^ percentile) before fixation, dimensions were 15.5 × 15 cm × 2 to 3.1 cm thickness. Umbilical cord (length: 16 cm) and membranes showed a normal morphology. After sectioning, focal and small whitish areas were observed close to the chorial surface and multiple, small (largest dimension <1 cm) hemorrhagic areas were also identified. Microscopically, this case showed morphological features consistent with intervillous hematomas (Fig. 2A) and microvascular thrombosis with intimal proliferations both in decidual vessels and villi (Fig. 2B). Significant foci of lymphocytic inflammation (CD8-positive) were present, involving the decidua and multiple villi (Fig. 2C–2D), findings consistent with chronic villitis (low grade, multifocal) [37]. Villous maturation was as expected for the gestational age.

#### Case 3

Placenta weight was 460 g (<3^rd^ percentile) prior to formalin fixation, dimensions: 14 × 14 cm × 2 to 3 cm thickness. Umbilical cord morphology was normal and insertion central, length: 28 cm. Membranes were translucent. Sectioning showed focal whitish areas (largest dimension: 8 mm). Histological features included foci of villous/intervillous lymphocytic inflammation involving both the maternal and the fetal sides as well the decidua; prevalence of CD8-positive cells was observed. Similar to the previous cases, these findings were consistent with chronic villitis (low grade, multifocal) [37]. Features suggestive of decidual vasculopathy and of chronic malperfusion were also present (thrombo-hemorrhagic alterations in decidual vessels, villous infarction, trophoblastic loss and focal thrombi). Villous maturation was within normal range with just focal chorangiosis.

#### Case 4

The first placenta weighted 410 g after formalin fixation, dimensions were 16 × 14 cm with a thickness ranging 1 to 3 cm. Umbilical cord length was 16 cm with a normal morphology and central insertion. Membranes presented a greenish discoloration and had a normal insertion. Sectioning showed extensive hemorrhagic subchorial areas consistent with partial placental abruption. Histological examination confirmed the presence of hemorrhagic areas located below the fetal surface, associated with chronic hypoxia signs (intervillous necrotic areas with focal calcifications). Accelerated villous maturation was also focally present. Decidual morphology was normal.

The second placenta weight was 235 g after fixation and dimensions were 14 × 15 cm with thickness ranging between 0.5 and 2.5 cm. Umbilical cord insertion was paracentral, and length was 20 cm. Membranes showed a greenish discoloration similar to the first placenta. Microscopically, features consistent with accelerated villous maturation were noted. No other abnormalities were present.

Placental weights were among the 10^th^–25^th^ percentiles (considering the sum of both placentas for twin pregnancies) [3].

#### Case 5

**Fig. 3:**
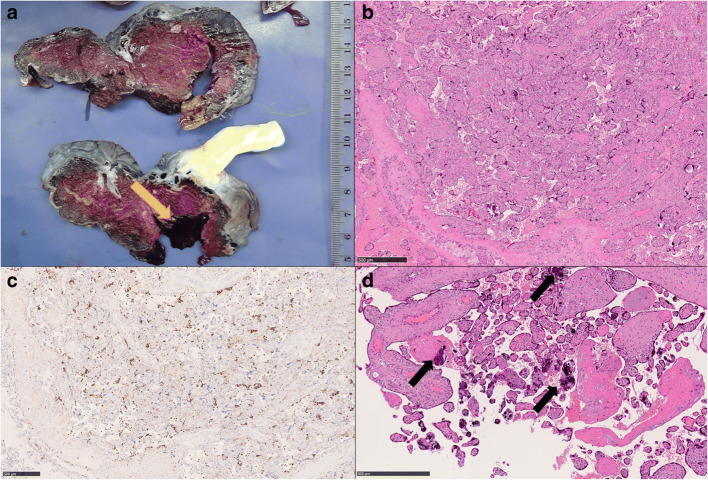
Pathological findings of Case 5. Focal placental abruption was noted during grossing (arrow) (**A**). Diffuse intervillous histiocytic infiltration was present consistently with the diagnosis of chronic histiocytic intervillositis (**B**: HE, 40X; **C**: anti-CD68 IHC, 40X). Villous agglutination with loss of trophoblasts and microcalcification was also observed (arrows) (**D**: HE, 70X)

Placenta weight was 370 g (10^th^–25^th^ percentiles) after fixation, dimensions: 15 × 12 cm with a thickness ranging between 1 to 3.2 cm. Umbilical cord length was 18 cm with a paracentral insertion. Membranes appearance was normal. After sectioning, focal abruption was observed (Fig. 3A). Histological examination showed intervillous histiocytic infiltration (CD68-positive) with perivillous fibrin deposition (Fig. 3B–3C), a finding consistent with chronic histiocytic intervillositis. Areas showing villous agglutination, loss of trophoblasts and focal microcalcifications were also observed (Fig. 3D). Focal features of accelerated villous maturation were also present.

#### Case 6

Placenta weighted 490 g (3^rd^–10^th^ percentiles) before fixation, dimensions: 17 × 17 cm with a thickness ranging between 1 and 3 cm. Placental macroscopic features were normal with translucent membranes. Umbilical cord length was 50 cm, with normal morphology and central insertion. The histological examination showed irregular villous maturation with focal chorangiosis, intervillous infarction and villous calcifications.

#### Case 7

Placenta weight before fixation was 400 g (<3^rd^ percentile), while its dimensions were 16 × 13 × 1 to 4 cm thickness. Membranes were translucent. The umbilical cord exhibited a normal morphology and marginal insertion with a total length of 18 cm. At sectioning, a focal congestion area was observed. Microscopically, features suggestive of umbilical blood flow restriction, like widespread chorangiosis, avascular villi with sclerosis and vascular stenosis together with hypertrophy of stem villi were noticed.

#### Case 8

Before fixation placenta weighted 520 g (10^th^–25^th^ percentiles), with total dimensions of 16 × 14 cm × 2 to 3.5 cm thickness. Membranes were yellowish and umbilical cord showed normal morphology and marginal insertion, with a length of 37 cm. No abnormalities were microscopically observed, except for focal villous hypermaturation and focal villous agglutination.

#### Case 9

This placenta was monochorionic diamniotic, with a total weight of 665 g before fixation, (3^rd^–10^th^ percentiles for twin deliveries) and dimensions of 19 × 16 cm with a thickness ranging between 3 and 4.5 cm. Membranes were translucent. The two umbilical cords were 23 and 20 cm in length, both hypocoiled and with marginal insertion. The first was characterized by focal edema. Histological examination showed a lymphocytic infiltration consistent with chronic villitis (low grade, multifocal). Accelerated villous maturation associated with focal hypoplasia and increase of syncytial nodes were also detectable. Features suggestive of fetal thrombotic vasculopathy like focal villous sclerosis were observed. Focal hemorrhagic area within basal decidua consistent with recent abruption was noted.

#### Case 10

Placenta weighted 440 g (3^rd^–10^th^ percentiles) before fixation, with dimensions 16 × 15 cm × 1 to 3 cm thickness. Membranes were translucent. Umbilical cord, with normal morphology and marginal insertion, was long 40 cm. Microscopically, this case showed villous hypoplasia and focal sclerosis, a finding consistent with previous placental infarction.

### Comparison with pre-COVID-19 control cohorts

To ascertain the specificity of the observed alterations, a control cohort of 58 pre-COVID-19 placentas was selected among placentas examined between March and July 2019, and this cohort was compared with COVID-19 placentas (Table 3). General characteristics were similar between the two groups (*p* > 0.05) except for the rate of previous cesarean sections which was higher in COVID-19 cases (*p* = 0.003). Regarding the observed pathological alterations, the incidence of VUE (*p* < 0.001), chronic deciduitis (*p* = 0.023), microvascular thrombosis (*p* = 0.003), presence of infarction areas (*p* = 0.047) and of accelerated villous maturation (*p* = 0.005) showed higher frequencies in placentas delivered by women with COVID-19.

**Table 3:** Comparison of general characteristics and pathological findings between placentas delivered by women with COVID-19 and the pre-COVID-19 control cohort

General characteristics	COVID-19 (*n* = 11) Median (range)	Control (*n* = 58) Median (range)	*p* value	Pathological findings	COVID-19 (*n* = 11)	Control (*n* = 58)	*p* value
Maternal age	33.5 (25–43)	34 (18–43)	0.772	Chronic villitis/VUE	4	0	**<0.001**
Gestational age	37 (30–40)	37 (30–41)	0.313	Histiocytic intervillositis	1	0	0.159
Placental weight	440 (235–665)	482 (260–1005)	0.378	Acute villitis	0	2	1.000
Fetal weight	2586 (950–3530)	2630 (1380–4920)	0.155	Chronic deciduitis	2	0	**0.023**
Previous cesarean section(s)	7	10	**0.003**	Acute deciduitis	0	5	0.585
				Choramniotitis	0	7	0.587
				Decidual microvascular thrombosis	3	0	**0.003**
				Intervillous hemorrhage	3	5	0.109
				Placental infarction areas	3	3	**0.047**
				Intervillous thrombi/fibrin deposition	3	9	0.390
				Chorangiosis	3	7	0.192
				Accelerated villous maturation	4	2	**0.005**
				Delayed villous maturation	1	12	0.676
				Distal villous hypoplasia	1	0	0.159
				Villous edema	0	2	1.000

Considering the significant difference in terms of previous cesarean sections, the COVID-19 series was compared with a further pre-COVID-19 control series with this characteristic (*n* = 28). Characteristics of this cohort are reported in Table 4. Only differences in terms of presence of chronic villitis/VUE (*p* = 0.035) and of accelerated villous maturation (*p* = 0.019) retained their statistically significance.

**Table 4:** Comparison of general characteristics and pathological findings between placentas delivered by women with COVID-19 who had previous cesarean section(s) and a pre-COVID-19 control cohort of placentas delivered by women who had previous cesarean section(s)

General characteristics	COVID-19 (*n* = 7) Median (range)	Control (*n* = 28) Median (range)	*p* value	Pathological findings	COVID-19 (*n* = 7)	Control (*n* = 28)	*p* value
Maternal age	36 (25–43)	34 (26–47)	0.238	Chronic villitis/VUE	2	0	**0.035**
Gestational age	35 (30–38)	37.5 (32–40)	**0.0002**	Histiocytic intervillositis	1	0	0.200
Placental weight	410 (235–665)	489 (236–820)	0.1738	Acute villitis	0	1	0.800
Fetal weight	2284.5 (950–3130)	2890 (1830–4160)	0.09492	Chronic deciduitis	1	0	0.200
				Acute deciduitis	0	0	-
				Choramniotitis	0	1	0.800
				Decidual microvascular thrombosis	1	0	0.200
				Intervillous hemorrhage	1	0	0.200
				Placental infarction areas	1	1	0.365
				Intervillous thrombi/fibrin deposition	2	2	0.171
				Chorangiosis	1	3	0.609
				Accelerated villous maturation	3	1	**0.019**
				Delayed villous maturation	0	6	0.232
				Distal villous hypoplasia	1	1	0.365
				Villous edema	0	2	0.635

## Discussion

In this study we report the pathological findings observed in eleven consecutive placentas delivered by ten women affected by COVID-19. Inflammatory and thrombo-hemorrhagic alterations were the most frequent and peculiar pathological changes we observed in our series compared to control samples, but only chronic villitis/VUE and accelerated villous maturation retained statistical significance after restricting the analysis to placentas delivered after a previous cesarean section.

In 5 cases we found microscopic signs of inflammation involving the decidua and the chorial villi: four out of five cases showed a CD8-positive T cell lymphocytic infiltrate, while in the remaining case a chronic histiocytic intervillositis was identified. None of these five patients had comorbidities, other than SARS-CoV-2 infection, which could justify the presence of placental inflammation. The presence of an inflammatory lymphocytic infiltrate (cytotoxic CD8+ T cell lymphocytes in particular, as observed in our series) and of villous damage is consistent with the diagnosis of chronic villitis [10, 39]. Chronic villitis etiology can be related to an undergoing infection, although in most cases, it is not possible to identify the specific etiopathogenetic agent: these cases are defined as “villitis of unknown etiology” (VUE) and can be found in 2–33.8% of placentas [38]. Compared to our control cohort, COVID-19 positive cases had a statistically significant higher frequency of this condition (*p* < 0.001).

Viruses are the most frequent cause of chronic villitis secondary to maternal infections: varicella zoster, herpes simplex virus, cytomegalovirus, and influenza A/H1N1, have been found to be associated with this condition [4, 9, 45, 56]. Little is known about the association between placental pathology and coronavirus infection. Seven placentas from coronavirus-infected women during the 2002–2004 SARS outbreak have been previously described [49]: histological examination found no abnormalities in 2/7, an increase in subchorionic and intervillous fibrin in 3/7, and massive thrombotic vasculopathy associated with intra-uterine growth restriction (IUGR) in the remaining 2/7 cases. No signs of chronic villitis were detected in any case and the same is true for MERS infections [36]. Moreover, considering experiences derived from influenza A/H1N1 infections in pregnancy, characterized by the activation of cellular immune response with release of inflammatory cytokines that lead to indirect placental damage [45], it stands to reason that SARS-CoV-2 may play an equivalent role in these histological examinations, because of intrinsic active virus replication and/or through indirect activity of inflammatory cytokines [43].

In the remaining case with inflammatory abnormalities, features consistent with chronic histiocytic intervillositis were identified. This rare entity is usually associated with maternal immunologic conditions like systemic lupus erythematosus, lupus anticoagulant and antiphospholipid syndrome [44, 46], while this patient’s significant comorbidities (chronic hypertension, severe obesity, and gestational diabetes) are not. Despite the different types of inflammatory infiltrate (histiocytic versus lymphocytic) and of etiologies (chronic histiocytic intervillositis is usually not related to viral infections); this pathological entity is frequently associated with chronic villitis (30 to 50% of cases), supporting a potential overlap in terms of involved immunologic pathways [41, 50]. Moreover, alveolar histiocytic infiltration is a frequent autoptic hallmark of SARS-CoV-2 infection [15]. Of note, despite its rarity, other cases of histiocytic intervillositis have been reported in placentas delivered by a SARS-CoV-2-positive women [34, 47].

Regarding the thrombo-hemorrhagic alterations, decidual microvascular thrombi were present in 3 cases, a significantly different frequency compared to the control cohort (*p* = 0.003) [15]. The relationship between COVID-19 and systemic coagulopathy with a thrombo-inflammatory state has been well documented [53, 54] and placental thromboses were also described in two cases of SARS-CoV during the previous outbreak as previously listed. Severe endothelial damage has also been described in lungs from COVID-19 patients associated with thrombosis and microangiopathy [55]. Placental microthrombosis may also be associated with antiphospholipid syndrome [42], but no patient in our series had a medical history or comorbidities suggesting the presence of antiphospholipid antibodies. Other findings, like placental hematomas, are common findings and probably unrelated to the SARS-CoV-2 infection. Concerning other thrombo-hemorrhagic findings like placental abruptions, an association between this condition and thrombophilia has been suggested [56] and an episode of placental abruption in a twin pregnancy complicated by COVID-19 was reported [57]. Features consistent with maternal vascular malperfusion, like accelerated villous maturation, were also more frequently observed within our series compared to the control cohort, but this hallmark can be secondary to multiple physiological and pathological conditions like twin pregnancy, hypertension, smoking and pre-eclampsia.

Regarding the previously reported series of placentas delivered by women with SARS-CoV-2 infection, no specific findings have been reported so far [6, 22, 31, 33, 47, 48, 53, 54]. In particular, Hecht et al. examined 19 placentas and found no characteristic histopathological features. This study is specifically relevant because a comprehensive analysis of both SARS-CoV-2 potential receptor/cofactor and viral RNA was performed. ACE2 was found to be commonly expressed by the syncytiotrophoblast, the cytotrophoblast and by scattered extravillous trophoblast. Strong positivity was also observed in maternal vessels. Conversely, TMPRSS2 was rarely and weakly positive in the villous endothelium, while the syncytiotrophoblast of one sample only showed a weak and patchy positivity. Regarding viral presence, SARS-CoV-2 RNA was detected in 2 out of 19 samples (one in the syncytiotrophoblast and one, focally, in both the syncytiotrophoblast and the cytotrophoblast) [33]. Viral RNA was also rarely present in the maternal endothelium within the decidua parietalis. These data support that direct placental infection by SARS-CoV-2 is a possible, although rare event. Evidence of potential placental infection by SARS-CoV-2 has also been confirmed by multiple assays, including electron microscopy [1, 55, 58].

Concerning the comparison of histopathological findings with control cohorts, Hecht et al. compared their COVID-19 placenta samples with both a series of “normal” (uncomplicated term deliveries) and “pathologic” controls (placentas of neonates with a clinical diagnosis of hypoxic ischemic encephalopathy). Interestingly, although the incidence of specific alterations like fetal vascular malperfusion (15%) was higher in COVID-19 placentas compared with the “normal” controls (5%), it was similar to the incidence in the “abnormal” cohort (17%). This finding is similar to ours: despite that the frequencies of multiple histopathological alterations were initially significantly different when comparing them with a general control cohort, only a few of them retained their significance after adjusting for a potential confounding factor (a previous cesarean section). Although the sample size reduction could have played a role, this result highlights the importance of taking into account potential confounding factors before suggesting a causal relationship. For example, abnormal placental implantation is another potential confounding variable which should be considered in future studies. In our cohort, only one case presented this finding (Case 10 – Marginal placenta previa).

Nevertheless, we detected significantly higher frequencies of chronic villitis/VUE and accelerated villous maturation even after accounting for a previous cesarean section. Recently, also Patberg et al. observed a higher incidence of villitis of unknown etiology (20.8 versus 7.1%) compared with a control group of term patients without COVID-19 [51]. This result was also confirmed in a subgroup analysis comparing asymptomatic COVID-19 patients with negative controls, but it did not reach statistical significance in a multivariable comparison adjusting for multiple clinical characteristics. This finding further enhances the importance of analyzing larger series of cases, possibly through dedicated meta-analyses, and adjusting for the effect of potential confounders. Regarding vascular/thrombotic alterations, 2 out of the 3 decidual microvascular thromboses we observed among COVID-19 cases were present in placentas delivered by women who had no previous cesarean section and thus they were independent of this potentially confounding variable. Therefore, the lack of significance observed by restricting the analysis to COVID-19 placentas delivered after a previous cesarean section could be at least partially due to sample size reduction.

Concerning our knowledge about the clinical outcomes of pregnancy in COVID-19 patients, Chen et al. [17] reported the outcomes of a relatively large series of pregnant women affected by COVID-19 observing a high rate (14/68, 21%) of preterm delivery. A systematic review evaluating 41 pregnancies affected by COVID-19 confirmed this observation, reporting preterm birth (<37 weeks) in above 40% of pregnancies [25]. Finally, even the largest systematic review available on 441 pregnant patients with COVID-19 also found that preterm birth is the most common adverse neonatal outcome (reported in the 21% of women who delivered) [29]. However, it should be noted that this higher rate of preterm deliveries could be at least partially explained by a different attitude during the early months of the pandemic favoring early delivery by cesarean section because of fear of potential COVID-19 complications.

Finally, the limited sample size and the intrinsically unspecific etiology of most of the observed placental alterations are the main limitations of our study as well of many of those published so far. Larger, multi-institutional prospective studies taking into account the whole set of variables related to SARS-CoV-2 infection (timing, severity, duration…), placental histopathological findings, potential confounders and materno-fetal outcomes are warranted to provide a definitive assessment of the relationship between COVID-19 and pregnancy.
